# Discovering interlinkages between major cryptocurrencies using high-frequency data: new evidence from COVID-19 pandemic

**DOI:** 10.1186/s40854-020-00213-1

**Published:** 2020-11-09

**Authors:** Imran Yousaf, Shoaib Ali

**Affiliations:** Air University School of Management, Air University, Islamabad, Pakistan

**Keywords:** Return spillover, Volatility spillover, Cryptocurrencies, Optimal weights, Hedge ratios, Hedging effectiveness, COVID-19, C58, G01, G11, G12

## Abstract

Through the application of the VAR-AGARCH model to intra-day data for three cryptocurrencies (Bitcoin, Ethereum, and Litecoin), this study examines the return and volatility spillover between these cryptocurrencies during the pre-COVID-19 period and the COVID-19 period. We also estimate the optimal weights, hedge ratios, and hedging effectiveness during both sample periods. We find that the return spillovers vary across the two periods for the Bitcoin–Ethereum, Bitcoin–Litecoin, and Ethereum–Litecoin pairs. However, the volatility transmissions are found to be different during the two sample periods for the Bitcoin–Ethereum and Bitcoin–Litecoin pairs. The constant conditional correlations between all pairs of cryptocurrencies are observed to be higher during the COVID-19 period compared to the pre-COVID-19 period. Based on optimal weights, investors are advised to decrease their investments (a) in Bitcoin for the portfolios of Bitcoin/Ethereum and Bitcoin/Litecoin and (b) in Ethereum for the portfolios of Ethereum/Litecoin during the COVID-19 period. All hedge ratios are found to be higher during the COVID-19 period, implying a higher hedging cost compared to the pre-COVID-19 period. Last, the hedging effectiveness is higher during the COVID-19 period compared to the pre-COVID-19 period. Overall, these findings provide useful information to portfolio managers and policymakers regarding portfolio diversification, hedging, forecasting, and risk management.

## Introduction

Thecryptocurrency market, a new asset class, has attracted significant attention from researchers, investors, policymakers, and governments in recent years (Makarov and Schoar 2020; Nasir et al. 2019). The size of the cryptocurrency market is continuously increasing due to (a) the decline in public trust toward the central banking system after the global financial crisis (Weber 2016), (b) the fourth industrial revolution and use of smart technologies, (c) its acceptance as legal currency in different countries, and (d) its acceptance by large companies like Facebook, Microsoft, Shopify, JPMorgan, and Tesla.^1^ Therefore, it is important to understand the dynamics of the cryptocurrency market, especially the interlinkages between the cryptocurrencies during the COVID-19 crisis. If, for example, volatility is transmitted from one cryptocurrency to another during the crisis period, then portfolio managers need to adjust their asset allocation to diversify risk, and financial policymakers need to adapt their policies in order to mitigate the contagion-related risk. The time-varying return and volatility linkages between different cryptocurrencies, especially during a crisis, have important implications for asset allocations, option pricing, and risk management (Kou et al. 2014; Caporin and Malik 2020).

In the related literature, numerous studies have examined the return/volatility spillover between different cryptocurrencies (Chu et al. 2017; Yi et al. 2018; Koutmos 2018; Baur and Dimpfl 2018; Ji et al. 2019; Katsiampa 2019; Katsiampa et al. 2019a, b; Canh et al. 2019; Beneki et al. 2019; Liu and Serletis 2019). For example, Yi et al. (2018) analyze the volatility connectedness between the 52 cryptocurrencies and find a volatility transmission from Bitcoin to other cryptocurrencies. Several other cryptocurrencies also transmit strong volatility effects; thus, Bitcoin is not the dominant transmitter of volatility to other cryptocurrencies. Koutmos (2018) examines the return and volatility transmission between the 18 major cryptocurrencies by using the approach of Diebold and Yilmaz (2009). Bitcoin is reported as the main transmitter of return and volatility effects to the other cryptocurrencies. Katsiampa (2019) employs the diagonal BEKK model and finds significant volatility co-movement between Bitcoin and Ethereum. Ji et al. (2019) study the return and volatility transmissions across six major cryptocurrencies (Bitcoin, Ethereum, Ripple, Litecoin, Stellar, and Dash) using the approach of Diebold and Yilmaz (2012) and find that Bitcoin and Litecoin are the net transmitters of return and volatility effects to the other cryptocurrencies. However, Ethereum, the second-largest currency, is the net recipient of the spillovers. Katsiampa et al. (2019a) uses the BEKK-MGARCH model to examine the shock and volatility transmission between three leading cryptocurrencies (Bitcoin, Ethereum, and Litecoin) and finds a bidirectional shock transmission between the pairs of Bitcoin–Ethereum and Bitcoin–Litecoin. Moreover, bidirectional volatility transmissions are observed between the Bitcoin–Ethereum, Bitcoin–Litecoin, and Ethereum–Litecoin pairs. Canh et al. (2019) investigate volatility dynamics across the seven major cryptocurrencies by employing the DCC-MGARCH model and find significant volatility transmission between all cryptocurrencies. Liu and Serletis (2019) employ the GARCH in mean model and find significant shock and volatility transmission between Bitcoin, Ethereum, and Litecoin. Beneki et al. (2019) apply the BEKK-GARCH technique to investigate the volatility transmission between Bitcoin and Ethereum. They find a unidirectional volatility spillover from Ethereum to Bitcoin. Based on the literature mentioned above, we noticed that none of the studies examined the spillovers between the cryptocurrency market during a crisis period. During various crises, many studies have examined the return/volatility spillover between different asset classes, for example, equity, bond, and commodity (Chen et al. 2002; Forbes and Rigobon 2002; Diebold and Yilmaz 2009; Aloui et al. 2011; Bekaert et al. 2014), but none have investigated cryptocurrencies. Hence, this study will address this literature gap.

The contribution of our study to the literature on cryptocurrencies is four-fold. First, this study investigates the return and volatility spillover between the cryptocurrencies during crisis (COVID-19) and pre-crisis (pre-COVID-19) periods. The reason for selecting the COVID-19 crisis is that almost all financial markets declined sharply worldwide, including stock, bond, commodity, energy, and cryptocurrency markets. Here are few glimpses of the fall of big markets during COVID-19. The Bitcoin price was down 19 percent on 23 March 2020 from its price on 01 January 2020. Moreover, the largest one-day fall in the price of Bitcoin was 36% on 13 March 2020. The S&P 500 and DJIA indices were down 33 percent and 36 percent, respectively, on 23 March 2020 from their peaks on 19 February 2020. The price for West Texas Intermediate (WTI) crude fell to an unbelievable $37.63 a barrel on 20 April 2020,^2^ and the China Manufacturing Purchasing Manager’s Index (PMI) was down 33% in February 2020.^3^ As cryptocurrencies have also been affected by COVID-19, the findings on spillovers can provide useful insights to crypto investors regarding portfolio and risk management during the COVID-19 pandemic.

Second, we estimate the return and volatility spillover using the VAR-AGARCH approach, proposed by McAleer et al. (2009). Previous studies have used various models/approaches, including the diagonal BEKK model, BEKK-MGARCH model, DCC-GARCH model, and the approach of Diebold and Yilmaz. While several studies have used the VAR-GARCH and VAR-AGARCH model to estimate spillover between different asset classes (Arouri et al. 2012; Jouini 2013; Yousaf and Hassan 2019), but no previous study has applied the VAR-AGARCH model to estimate return and volatility spillover between cryptocurrencies. The model used in this study includes the constant conditional correlation (CCC-GARCH) model of Bollerslev (1990) as a special case. This model is selected for three reasons: (a) the most commonly used multivariate models, like the BEKK and DCC-GARCH models, often suffer from unreasonable parameter estimates and data convergence problems (Bouri 2015). The VAR-AGARCH model overcomes these problems regarding parameters and convergence. (b) It incorporates asymmetry in the model, and (c) this model also calculates the optimal weights and hedge ratios.

Third, we use high frequency (hourly) data to examine linkages between the cryptocurrencies, which provides a better and deeper insight to crypto investors. In the above-mentioned literature, all studies use daily data to study linkages between cryptocurrencies, except Katsiampa et al. (2019b). Finally, we also estimate the optimal weights and hedge ratios for pairs of cryptocurrencies during the pre-COVID-19 and COVID-19 periods in order to provide useful insights to portfolio managers regarding asset allocation and efficient portfolio management during crisis and non-crisis periods.

The rest of the paper is organized as follows: Second section describes the “Methodology”, and third section provides the “Data and preliminary analysis”. Fourth section reports the “Empirical findings”, and fifth section “Conclude” the paper.

## Methodology

In this section, we first present the VAR-AGARCH model and then describe the method used to calculate optimal weights, hedge ratios, and hedging effectiveness for the pairs of cryptocurrencies.

### VAR-AGARCH model

McAleer et al. (2009) proposed the multivariate VAR-AGARCH Model to estimate the return and volatility transmission between the different series. The VAR-AGARCH model assumes that positive or negative shocks do not have the same impact on conditional variance, and it incorporates asymmetry in the model. For multiple series, the VAR-AGARCH model has the following specifications for the conditional mean equation:

1 $$R_{t} = \mu + \emptyset R_{t - 1} + e_{t} \,{\text{with}}\,e_{t} = D_{t}^{1/2} \eta_{t} ,$$

in which *Rt* represents a 3 × 1 vector of daily returns of x, y, and z cryptocurrencies at time t; $\mu$ denotes a 3 × 1 vector of constants; $$\emptyset = \left( {\begin{array}{*{20}c} {\emptyset_{11} } & {\emptyset_{12} } & {\emptyset_{13} } \\ {\emptyset_{21} } & {\emptyset_{22} } & {\emptyset_{23} } \\ {\emptyset_{31} } & {\emptyset_{32} } & {\emptyset_{33} } \\ \end{array} } \right)$$ is a 3 × 3 matrix of parameters measuring the impacts of own lagged and cross mean transmissions between three series; $e_{t}$ is the residual of the mean equation for the three series of cryptocurrency returns at time t; $\eta_{t}$ indicates a 3 × 1 vector of independently and identically distributed random vectors; and $D_{t}^{1/2}$ = *diag* ($\sqrt {h_{t}^{x} }$, $\sqrt {h_{t}^{y} }$, $\sqrt {h_{t}^{z} }$), where $h_{t}^{x}$, $h_{t}^{y}$, and $h_{t}^{z}$ represent the conditional variances of the returns for cryptocurrency x, y, and z, respectively. The specifications of the VARMA–AGARCH model are given as follows:

2 $$h_{it} = c_{ii} + \mathop \sum \limits_{j = 1}^{n} a_{ij} e_{jt - 1}^{2} + \mathop \sum \limits_{j = 1}^{n} b_{ij} h_{jt - 1} + d_{i} e_{it - 1}^{2} I\left( {e_{it - 1} } \right)$$

where $e_{jt - 1}^{2}$ and $h_{jt - 1}$ capture the ARCH and GARCH effects, respectively. Equation (2) implies that the conditional variance of each market depends upon their own past shock and volatility as well as on the past shock and volatility of other markets. The indicator function $I\left( {e_{it - 1} } \right)$ is equal to one if $e_{it - 1} < 0$, and 0 zero otherwise. For this specification, a positive value for d means that negative residuals tend to increase the variance more than positive ones. The asymmetric effect is designed to capture the characteristic in which an unexpected drop in asset prices tends to increase volatility more than an unexpected increase in asset prices of the same magnitude (Chao et al. 2020; Wang et al. 2020; Shen et al. 2020).

Furthermore, the conditional covariance between different cryptocurrencies can be estimated as follows:

3 $$h_{t}^{x,y} = p \times \sqrt {h_{t}^{x} } \times \sqrt {h_{t}^{y} } .$$

In the above equation, $$h_{t}^{x,y}$$ refers to the conditional covariance between the returns of two cryptocurrencies ($$x, y)$$ at time t. Moreover, $$p$$ indicates the constant conditional correlation between the returns of two cryptocurrencies ($$x, y)$$.

### Optimal weights and hedge ratios

The estimates of the VAR-AGARCH model can be used to calculate optimal portfolio weights. This study follows Kroner and Ng (1998) to calculate the optimal portfolio weights for the pairs of cryptocurrencies (x, y):

4 $$\begin{aligned} w_{xy,t} & = \frac{{h_{y,t} - h_{xy,t } }}{{h_{x,t} - 2h_{xy,t} + h_{y,t} }} \\ w_{xy,t} & = \left\{ {\begin{array}{*{20}l} {0,} \hfill & {If\quad W_{xy,t} < 0} \hfill \\ {w_{xy,t} ,} \hfill & {If\quad 0 \le w_{xy,t} \le 1} \hfill \\ {1,} \hfill & {If\quad w_{xy,t} > 1} \hfill \\ \end{array} } \right., \\ \end{aligned}$$

where $w_{xy,t}$ is the weight of cryptocurrency ($x$) in a one-dollar portfolio of cryptocurrency (x) and cryptocurrency ($y$) at time t; $h_{xy,t}$ is the conditional covariance between the two cryptocurrencies; $h_{x,t}$ and $h_{y,t}$ are the conditional variance of cryptocurrency ($x$) and cryptocurrency ($y$), respectively; and 1 − $w_{xy,t}$ is the weight of cryptocurrency ($y$) in a dollar one portfolio of cryptocurrency ($x$) and cryptocurrency ($y$).

It is also essential to estimate the risk-minimizing optimal hedge ratios for a portfolio of different pairs of cryptocurrencies. The estimates of the VAR-AGARCH model can also be used to calculate optimal hedge ratios. This study follows Kroner and Sultan (1993) to calculate the optimal hedge ratios.

5 $$\beta_{xy,t} = \frac{{h_{xy,t} }}{{h_{y,t} }},$$

where $\beta_{xy,t}$ represents the hedge ratio. This shows that a short position in cryptocurrency ($y$) can hedge a long position in cryptocurrency ($x$).

### Hedging effectiveness

Hedging effectiveness is estimated to compare the performance of optimal portfolios. If the hedging effectiveness is 1, then it represents a perfect hedge and vice versa. Thus, a higher hedging effectiveness score shows a higher risk reduction. Following Ku et al. (2007) and Pan et al. (2014), this study estimates hedging effectiveness (HE) as follows:

6 $$HE = \frac{{variance_{Unhedged} - variance_{hedged} }}{{variance_{Unhedged} }},$$

where $variance_{Unhedged}$ represents the variance of the unhedged portfolio (only x asset) returns, and $variance_{hedged}$ indicates the variation in the returns for the portfolio of x and y assets $Variance_{hedged} = h_{x,t} + \beta_{xy,t}^{2} . h_{y,t} - 2\beta_{xy,t} .h_{xy,t }$.

### Robustness check

In Eq. (3), $p$ is not time-varying, but the time-varying conditional correlation is a stylized fact in financial data. The dynamic correlation also plays an important role in the dynamic hedge ratio. Several studies have used the estimates of the VAR-BEKK-GARCH or VAR-BEKK-AGARCH model to calculate time-varying correlations and optimal hedge ratios (Lin et al. 2014; Lin 2017; Klein et al. 2018; Beneki et al. 2019; Belhassine 2020). Therefore, for robustness purposes, we also calculate time-varying correlations, optimal weights, hedge ratios, and hedging effectiveness using the VAR-BEKK-AGARCH model in this study.^4^ Lastly, RATS 10 software is used for all estimations in this study.

## Data and preliminary analysis

### Data

This study uses the hourly data of three cryptocurrencies (Bitcoin, Ethereum, and Litecoin), representing 76% (as of 01 April 2020) of market capitalization in the cryptocurrency market. We use two sample periods, the pre-COVID-19 period (hourly data from 03/10/2018 to 31/12/2019) and the COVID-19 period (hourly data from 01/01/2020 to 01/04/2020). Following Corbet et al. (2020) and Haroon and Rizvi (2020), we start the COVID-19 period from 01/01/2020. The data on cryptocurrency prices are taken from Bittrex, and the prices are listed in US dollars.

### Preliminary analysis

**Fig. 1:**
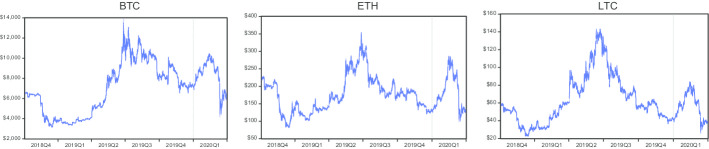
Hourly prices of cryptocurrencies

**Fig. 2:**
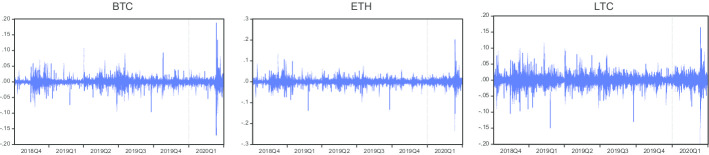
Hourly returns of cryptocurrencies

Figure 1 presents the hourly prices of Bitcoin (BTC), Ethereum (ETH), and Litecoin (LTC). The figure shows that the prices of these three cryptocurrencies decreased in 2018Q4, but then increased in 2019Q1 and 2019Q2. Prices of all cryptocurrencies again revealed a declining trend in 2019Q3 and 2019Q4, and then prices increased in the first half of 2020Q1 before decreasing in the second half of the quarter. The huge decline in prices indicates that COVID-19 adversely affected cryptocurrency prices during the second half of 2020Q1. Almost all currencies followed a similar trend during the reported six quarters. Figure 2 reveals the hourly returns of Bitcoin, Ethereum, and Litecoin. It shows the volatility clustering in returns of all cryptocurrencies in different quarters. However, peak volatilities can be observed in Bitcoin, Ethereum, and Litecoin during 2020Q1 (the COVID-19 period).

Table 1 presents the summary statistics of the returns of Bitcoin, Ethereum, and Litecoin during the pre-COVID-19 period (see Panel A) and COVID-19 period (see Panel B). The average returns of Bitcoin are positive in the pre-COVID-19 period, whereas they were highly negative during the COVID-19 period. This shows that Bitcoin has been highly and adversely affected by the COVID-19 global pandemic. In contrast, the mean returns of Ethereum are negative during the pre-COVID-19 period, whereas they are highly positive during COVID-19. Lastly, the mean returns of Litecoin are negative in both the pre-COVID-19 and COVID-19 periods.

**Table 1:** Descriptive statistics

	Mean	Maximum	Minimum	SD	Skewness	Kurtosis	Jarque–Bera	Q-stat	ARCH	ADF[C]	ADF[C+T]
*Panel A. Pre COVID-19*											
BTC	0.000009	0.107	− 0.096	0.008	0.041	28.743	301,467^a^	21.216^a^	120.06^a^	− 106.37^a^	− 106.37^a^
ETH	− 0.000050	0.129	− 0.138	0.010	− 0.209	27.573	274,772^a^	23.354^a^	76.898^a^	− 107.06^a^	− 107.06^a^
LTC	− 0.000033	0.115	− 0.150	0.012	− 0.095	18.213	105,302^a^	101.71^a^	172.94^a^	− 80.373^a^	− 80.376^a^

	Mean	Maximum	Minimum	SD	Skewness	Kurtosis	Jarque–Bera	Q-stat	ARCH	ADF-1	ADF-2
*Panel B. COVID-19*											
BTC	− 0.000059	0.188	− 0.172	0.012	− 0.877	82.750	582,223^a^	34.127^a^	95.741^a^	− 36.559^a^	− 36.566^a^
ETH	0.000011	0.201	− 0.232	0.014	− 1.713	70.278	415,236^a^	45.686^a^	60.630^a^	− 52.153^a^	− 52.165^a^
LTC	− 0.000030	0.164	− 0.196	0.015	− 0.648	32.614	80,397^a^	68.992^a^	155.18^a^	− 53.489^a^	− 53.501^a^

BTC, Bitcoin; ETH, Ethereum; LTC, Litecoin; ETC. Q-stat denotes the Ljung–Box Q-statistics. The ARCH test refers to the LM-ARCH test of Engle (1982). ADF[C] refers to the augmented dickey fuller test with constant. ADF[C+T] indicates the augmented dickey fuller test with constant and trend

^a^Indicate the statistical significance at 1%, 5% and 10% respectively

However, the unconditional volatility is lowest in Bitcoin and highest in Litecoin during both sample periods. In all three cryptocurrencies, the returns are skewed to the left (in most cases), kurtosis is significantly higher than 3, and the Jarque–Bera statistics reject the normality hypothesis. Moreover, the results also confirm the presence of autocorrelation and ARCH effects in the returns of all three cryptocurrencies during both sample periods. In addition, we apply the Augmented Dickey–Fuller (ADF) test to examine the stationarity and find all series to be significant, inferring that the returns of all three cryptocurrencies are stationary during both sample periods. Last, we apply the unit root test with break dates, and the results are available in Table 6 (Appendix).

Finally, Table 2 provides the correlation matrix for three pairs of cryptocurrencies, namely BTC-ETH, BTC-LTC, and ETH-LTC, in the pre-COVID-19 and COVID-19 periods. The correlations are positively significant and above 0.645 for all three pairs of cryptocurrencies during both sample periods. These correlations are consistent with the study by Katsiampa et al. (2019b), which finds a correlation above 0.717 for the pairs of BTC-ETH, BTC-LTC, and ETH-LTC using hourly returns data. Further, the unconditional correlations are found to be higher during the COVID-19 period compared to the pre-COVID-19 period.

**Table 2:** Correlation matrix

	Pre COVID-19				COVID-19		
	BTC	ETH	LTC		BTC	ETH	LTC
BTC	1			BTC	1		
ETH	0.782^a^	1		ETH	0.891^a^	1	
LTC	0.645^a^	0.710^a^	1	LTC	0.814^a^	0.853^a^	1

BTC, Bitcoin; ETH, Ethereum; LTC, Litecoin

^a^Indicate the statistical significance at 1%, 5% and 10% respectively

## Empirical results

### Return and volatility spillovers

To examine the return and volatility spillovers between Bitcoin, Ethereum, and Litecoin, we use the multivariate VAR-AGARCH model presented in Eqs. 1 and 2. The results are reported in Table 3. We notice a significant autocorrelation and ARCH effects for the returns of all three cryptocurrencies, as shown in Table 1; hence, we can employ a multivariate VAR-AGARCH model in our analysis.

**Table 3:** Estimates of multivariate VAR-AGARCH model for the Bitcoin, Ethereum, and Litecoin

	Pre COVID-19		COVID-19	
	Coefficient	*P *value	Coefficient	*P* value
*Panel A. Mean equation*				
$\mu_{1}$	− 0.000	0.380	0.000	0.349
$\emptyset_{11}$	− 0.171^a^	0.000	− 0.093^b^	0.019
$\emptyset_{12}$	− 0.018	0.501	0.076^c^	0.052
$\emptyset_{13}$	0.102^c^	0.072	0.083	0.150
$\mu_{2}$	− 0.000	0.277	0.000^c^	0.052
$\emptyset_{21}$	0.067 ^a^	0.000	0.018	0.499
$\emptyset_{22}$	− 0.081^a^	0.000	− 0.221^a^	0.000
$\emptyset_{23}$	0.182^a^	0.000	0.132^c^	0.094
$\mu_{3}$	− 0.000	0.323	0.000	0.249
$\emptyset_{31}$	0.017^c^	0.071	0.015	0.509
$\emptyset_{32}$	0.026^c^	0.052	0.043	0.223
$\emptyset_{33}$	− 0.225^a^	0.000	− 0.188^a^	0.000
*Panel B. Variance equation*				
$c_{1}$	0.000^a^	0.000	0.000^b^	0.012
$c_{2}$	0.001^a^	0.000	0.001^a^	0.000
$c_{3}$	0.000^a^	0.000	0.001^a^	0.000
$a_{11}$	0.073^b^	0.018	− 0.029^c^	0.070
$a_{12}$	0.014^b^	0.019	0.042^a^	0.000
$a_{13}$	0.027^a^	0.000	− 0.002^c^	0.055
$a_{21}$	0.011^c^	0.068	0.061^a^	0.000
$a_{22}$	0.045^a^	0.000	0.150^a^	0.000
$a_{23}$	0.060^a^	0.000	0.015^c^	0.061
$a_{31}$	0.002	0.293	− 0.065^a^	0.000
$a_{32}$	0.117^a^	0.001	0.083^a^	0.000
$a_{33}$	0.181^a^	0.000	0.063^a^	0.000
$b_{11}$	0.880^a^	0.000	0.995^a^	0.000
$b_{12}$	− 0.144^a^	0.000	0.037^a^	0.000
$b_{13}$	− 0.129^a^	0.000	0.025^b^	0.040
$b_{21}$	− 0.058^b^	0.032	− 0.035^c^	0.052
$b_{22}$	1.256^a^	0.000	0.874^a^	0.000
$b_{23}$	− 0.269^a^	0.000	− 0.019^c^	0.094
$b_{31}$	− 0.153	0.113	0.027^c^	0.079
$b_{32}$	1.028^a^	0.000	0.069^a^	0.000
$b_{33}$	0.860^a^	0.000	0.938^a^	0.000
$d_{1}$	− 0.035^b^	0.043	0.041^b^	0.018
$d_{2}$	− 0.016^c^	0.061	0.047^a^	0.001
$d_{3}$	− 0.017^c^	0.082	0.034^a^	0.006
*Panel C: Constant correlations*				
$p_{21}$	0.783^a^	0.000	0.860^a^	0.000
$p_{31}$	0.644^a^	0.000	0.802^a^	0.000
$p_{32}$	0.691^a^	0.000	0.831^a^	0.000
*Panel D: Robustness tests*				
Log L	11,912.2		24,025.6	
AIC	− 20.972		− 20.392	
SIC	− 20.850		− 19.918	
$Q_{1}$(20)	42.173^a^	0.003	36.776^b^	0.012
$Q_{2}$(20)	45.954^a^	0.000	36.444^b^	0.013
$Q_{3}$(20)	22.238	0.245	25.542	0.181
$Q_{1}^{2}$(20)	2.610	0.991	18.776	0.352
$Q_{2}^{2}$(20)	4.859	0.988	14.444	0.415
$Q_{3}^{2}$(20)	5.166	0.981	10.542	0.480

# of lags for VAR is decided using SIC and AIC criteria. JB, Q(20), and Q^2^(20) indicate the empirical statistics of Jarque–Bera test for normality, Ljung–Box Q statistics of order 20 for autocorrelation applied to the standardized residuals and squared standardized residuals, respectively. BTC, Bitcoin; ETH, Ethereum; LTC, Litecoin. Variable order is the Bitcoin (1), Ethereum (2), and Litecoin (3). In the mean equations, $\mu$ denotes the constant terms, whereas $\emptyset_{12}$ denotes the return spillover from Bitcoin to Ethereum. In the variance equation, 'c' denotes the constant terms, 'a' denotes the ARCH terms, and 'b' denotes the GARCH terms. In the variance equation, $a_{12}$ indicates the shock spillover from Bitcoin to Ethereum, whereas ${\text{b}}_{12}$ denotes the long-term volatility spillover from Bitcoin to Ethereum. $d_{1}$ is the asymmetric effect of the Bitcoin

^a,b,c^Indicate the statistical significance at 1%, 5% and 10% respectively

#### Return spillovers

Table 3 presents the return and volatility transmission between Bitcoin, Ethereum, and Litecoin during the pre-COVID-19 and COVID-19 periods. Referring to Panel A, the coefficients of own-mean spillover ($\emptyset_{11}$, $\emptyset_{22}$, and $\emptyset_{33}$) are significantly negative during both periods, indicating that the lagged returns inversely affect their current returns in Bitcoin, Ethereum, and Litecoin during both sample periods. These results are consistent with the findings of Liu and Serletis (2019), who find that own-mean spillovers are significant in Bitcoin, Ethereum, and Litecoin. These findings highlight the possibility of short-term predictions of current returns through past returns for Bitcoin, Ethereum, and Litecoin.

Regarding the return spillovers between Bitcoin and Ethereum ($\emptyset_{12} , \emptyset_{21}$) in the mean equation, the results indicate a unidirectional and positive return spillover from Ethereum to Bitcoin during the pre-COVID-19 period. These results are consistent with the findings of Liu and Serletis (2019), they find that the lagged returns of Ethereum significantly influence the current returns of Bitcoin. This implies that Ethereum returns were useful in forecasting Bitcoin returns in the pre-COVID-19 period. In contrast, the return spillover is found to be unidirectional and positive from Bitcoin to Ethereum during the COVID-19 period, suggesting that Bitcoin returns can be used to forecast Ethereum during the crisis. This indicates that when the Bitcoin returns decreased during the COVID-19 period, investors tended to decrease investment in Ethereum as well due to fear of huge losses, thus bidding down the price of Ethereum.

Based on the return spillovers between Bitcoin and Litecoin ($\emptyset_{13} , \emptyset_{31}$), the finding reveals a bidirectional and positive return transmission between Bitcoin and Litecoin during the pre-COVID-19 period. However, the return transmission is not significant between Bitcoin and Litecoin during the COVID-19 period. This implies that Litecoin (Bitcoin) returns cannot be used to forecast Bitcoin (Litecoin) returns during the COVID-19 period. Lastly, based on the return transmission between Ethereum and Litecoin ($\emptyset_{23} , \emptyset_{32}$), the results indicate a bidirectional and positive return spillover between Ethereum and Litecoin during the pre-COVID-19 period. Moreover, a unidirectional return transmission is observed from Ethereum to Litecoin during the COVID-19 period. This implies that Ethereum returns can be used to forecast Litecoin returns during the COVID-19 period.

#### Volatility spillovers

Regarding own-shock ($a_{11}$, $a_{22}$, and $a_{33}$) and own-volatility spillovers ($b_{11}$, $b_{22}$, and $b_{33}$), the findings show that the lagged shocks and volatility significantly and positively influence their current conditional volatility in Bitcoin, Ethereum, and Litecoin during both sample periods, except for Bitcoin during COVID-19. These results are in line with the findings of Katsiampa et al. (2019b). During COVID-19, the own-shock spillover is negative and significant in Bitcoin, suggesting that past shocks inversely affect the current volatility in Bitcoin in the COVID-19 period. Overall, the coefficients of past own-volatility are higher compared to the coefficients of past own-shocks, implying that past own-volatilities are a more important factor in predicting current volatilities compared to past own-shocks during both sample periods.

Based on cross-market shock spillover ($a_{12}$, $a_{13}$, $a_{21}$, $a_{23}$, $a_{31}$ and $a_{32}$), the results indicate that the shock spillover is positive and bidirectional for the pairs of Bitcoin–Ethereum and Ethereum–Litecoin during both sample periods. These results are similar to the findings of Katsiampa et al. (2019a), who find a bidirectional shock spillover between Bitcoin and Ethereum. In contrast, the shock transmission is positive and unidirectional from Bitcoin to Litecoin during pre-COVID-19 period, whereas it is negatively significant between Bitcoin and Litecoin during the COVID-19 period.

With regards to the volatility spillovers between Bitcoin and Ethereum ($b_{12}$ and $b_{21}$), the findings reveal a bidirectional and negative volatility spillover between Bitcoin and Ethereum during the pre-COVID-19 period. These results are in line with the findings of Katsiampa et al. (2019a), who find a bidirectional volatility transmission between Bitcoin and Ethereum. During the COVID-19 period, the volatility transmission is positive and significant from Bitcoin to Ethereum, whereas it is negatively significant from Ethereum to Bitcoin. As volatility transmission is bidirectional, these findings suggest that investors could not get the maximum benefit of diversification by making a portfolio of Bitcoin and Ethereum during either period. Regarding cross-market volatility spillover between Bitcoin and Litecoin ($b_{13}$ and $b_{31}$), the results indicate a unidirectional and negative volatility transmission from Bitcoin to Litecoin during the pre-COVID-19 period. However, the volatility spillover is bidirectional between Bitcoin and Litecoin during the COVID-19 period. Based on the cross-market volatility spillover ($b_{23}$ and $b_{32}$), the results indicate a bidirectional volatility transmission between Ethereum and Litecoin during both sample periods. These results are similar to the findings of Katsiampa et al. (2019a), who find bidirectional volatility transmission between Ethereum and Litecoin. Moreover, the volatility transmission is negative from Ethereum to Litecoin, whereas it is positive from Litecoin to Ethereum during both sample periods.

The asymmetric coefficients ($d_{1} , d_{2} , d_{3}$) of all three cryptocurrencies are significantly negative during pre-COVID-19 period, indicating that positive residuals tend to increase the variance more than negative ones.^5^ In other words, good news increased volatility more than bad news in the pre-COVID-19 period. In contrast, the asymmetric coefficients of cryptocurrencies are found to be positive and significant during the COVID-19 period, indicating that negative unexpected shocks increased volatility in cryptocurrencies more than positive shocks during the COVID-19 period. In other words, an unexpected drop in asset prices tends to increase volatility more than an unexpected increase in asset prices of the same magnitude. The increased volatility in response to negative shocks might be explained by the herding of investors (Yousaf et al. 2018; Wen et al. 2019), that is, the selling of cryptocurrencies due to the fear of loss after the huge decline in global business activity during the COVID-19 period.

Referring to Panel C, the constant conditional correlations ($p_{21} ,p_{31} ,p_{32}$) are significantly positive during both sample periods, consistent with the findings of Katsiampa et al. (2019b) and Canh et al. (2019). Moreover, correlations are observed to be higher during the COVID-19 period compared to the pre-COVID-19 period. This implies that cryptocurrencies are highly linked during the COVID-19 period. For robustness purposes, we also estimate the return and volatility spillovers between the cryptocurrencies after slightly changing the two sample periods; the results are reported in Table 7 (Appendix).^6^

### Optimal weights and hedge ratios-portfolio implications

Table 4 reports the optimal weights and hedge ratios for the pairs of BTC/ETH, BTC/LTC, and ETH/LTC during the pre-COVID-19 and COVID-19 periods. The findings reveal that the optimal weight is 0.84 for the pair of BTC/ETH during the pre-COVID-19 period, indicating that for a $1 portfolio of BTC-ETH, 84 cents should be invested in Bitcoin and the remaining 16 cents in Ethereum. Katsiampa (2019) also find that Bitcoin should outweigh Ethereum in terms of optimal portfolio weights. For a $1 portfolio BTC-LTC, investors should allocate 92 cents in Bitcoin during the pre-COVID-19 period, and investors should allocate 82 cents for Ethereum in a $1 portfolio of ETH-LTC during the pre-COVID-19 period. During COVID-19, for a $1 portfolio of BTC-ETH, investors should invest 82 cents in Bitcoin and the remaining 18 cents in Ethereum. For a $1 portfolio of BTC-LTC, investors should allocate 90 cents in Bitcoin and the remaining 10 cents in Litecoin during COVID-19. Last, investors should invest 80 cents in Ethereum for a $1 portfolio of ETH-LTC during the COVID-19 period.

**Table 4:** Optimal Weights and Hedge Ratios for pairs of cryptocurrencies

	BTC/ETH	BTC/LTC	ETH/LTC
Pre COVID-19 period			
$w_{t}$(*VAR-AGARCH*)	0.84	0.92	0.82
$w_{t}$(*VAR-BEKK-AGARCH*)	0.86	0.93	0.84
$\beta_{t}$(*VAR-AGARCH*)	0.62	0.41	0.55
$\beta_{t}$(*VAR-BEKK-AGARCH*)	0.62	0.41	0.55
COVID-19 period			
$w_{t}$(*VAR-AGARCH*)	0.82	0.90	0.80
$w_{t}$(*VAR-BEKK-AGARCH*)	0.84	0.90	0.79
$\beta_{t}$(*VAR-AGARCH*)	0.64	0.50	0.67
$\beta_{t}$(*VAR-BEKK-AGARCH*)	0.66	0.53	0.71

$w_{t}$ and $\beta_{t}$ refer to the optimal weights and hedge ratios, respectively

Overall, the optimal weights are found to be lower for the pairs of BTC/ETH and BTC/LTC during the COVID-19 period compared to the pre-COVID-19 period, suggesting that cryptocurrency investors should decrease their investments in Bitcoin for the BTC/ETH and BTC/LTC portfolios during the COVID-19 period. Moreover, the optimal weights are also found to be lower for the ETH/LTC pair during the COVID-19 period compared to the pre-COVID-19 period, implying that investors should reduce their asset allocation in Ethereum for the portfolio of ETH/LTC during the COVID-19 period. For robustness purposes, we also calculate the optimal weights using the VAR-BEKK-AGARCH model, which are given in Table 4.

Based on the optimal hedge ratios, the results indicate that the optimal hedge ratio is 0.62 for the BTC/ETH pair during the pre-COVID-19 period, indicating that a $1 long position in Bitcoin can be hedged for 62 cents with a short position in Ethereum. A 41-cent short position in Litecoin can hedge a $1 long position in Bitcoin during the pre-COVID-19 period. A $1 long position in Ethereum can be hedged for 55 cents with a short position in Litecoin during the pre-COVID-19 period. During the COVID-19 period, a $1 long position in Bitcoin can be hedged for 64 cents with a short position in Ethereum. A 50-cent short position in Litecoin can hedge a $1 long position in Ethereum during the COVID-19 period. Finally, a $1 long position Ethereum can be hedged for 67 cents with a short position in Litecoin during the COVID-19 period.

**Fig. 3:**
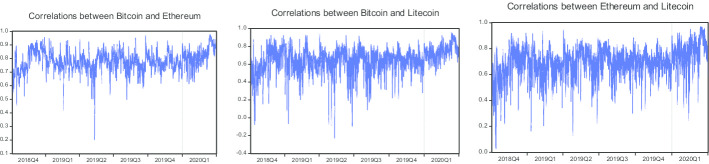
Time-varying correlations computed from the VAR-BEKK-AGARCH model

**Fig. 4:**
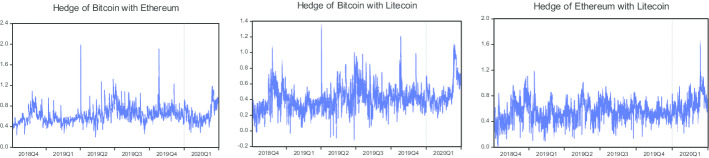
Time-varying Hedge ratios computed from the VAR-BEKK-AGARCH model

Overall, the optimal hedge ratios are higher for the pairs of BTC/ETH during the COVID-19 period compared to the pre-COVID-19 period, suggesting that more Ethereum is needed to minimize the risk of Bitcoin during the COVID-19 period compared to the pre-COVID-19 period. Moreover, the optimal hedge ratios are also observed to be higher for the pairs of BTC/LTC and ETH/LTC during the COVID-19 period than in the pre-COVID-19 period, suggesting that more Litecoin is needed to minimize the risk of Bitcoin and Ethereum during the COVID-19 period. For robustness purposes, we also estimate the optimal hedge ratios using the VAR-BEKK-AGARCH model, which are given in Table 4. Moreover, time-varying correlations and hedge ratios for all three pairs are available in Figs. 3 and 4.

### Hedging effectiveness

We also estimate the hedging effectiveness for the BTC/ETH, BTC/LTC, and ETH/LTC pairs during the pre-COVID-19 and COVID-19 periods (Table 5). We estimate hedging effectiveness by using the optimal weights and hedge ratios of two models, including the main models of VAR-AGARCH and VAR-BEKK-AGACH for robustness analysis. The results reveal that the risk-adjusted returns improve by constructing the portfolios of BTC-ETH, BTC-LTC, and ETH-LTC during both periods. For the portfolios of BTC-ETH, BTC-LTC, and ETH-LTC, the hedging effectiveness is higher during the COVID-19 period than the pre-COVID-19 period. Lastly, the hedging effectiveness of the VAR-AGARCH model is higher compared to the VAR-BEKK-AGARCH model during both sample periods.

**Table 5:** Hedging effectiveness (%) for the pairs of cryptocurrencies

	BTC/ETH	BTC/LTC	ETH/LTC
Pre COVID-19 period			
* HE* (*VAR-AGARCH*)	61.49	39.37	47.84
* HE* (*VAR-BEKK-AGARCH*)	57.10	35.76	46.55
COVID-19 period			
* HE* (*VAR-AGARCH*)	76.32	61.78	70.40
* HE* (*VAR-BEKK-AGARCH*)	68.88	57.29	65.85

## Conclusion

In this study, we apply the VAR-AGARCH model to intra-day data for three major cryptocurrencies, Bitcoin, Ethereum, and Litecoin, in order to examine the return and volatility spillovers between cryptocurrencies during the pre-COVID-19 and COVID-19 periods. We also estimate the optimal weights, hedge ratios, and hedging effectiveness between the pairs of cryptocurrencies during both periods.

The findings reveal a unidirectional return spillover from Ethereum to Bitcoin during the pre-COVID-19 period, and a unidirectional return spillover from Bitcoin to Ethereum during the COVID-19 period. This suggests that, in the short run, Bitcoin (Ethereum) returns can be used to forecast Ethereum (Bitcoin) returns during the COVID-19 period (pre-COVID-19 period). For the pair of Bitcoin and Litecoin, bidirectional return spillover is observed during the pre-COVID-19 period, whereas the return spillover is not significant during the COVID-19 period. For the pair of Ethereum and Litecoin, the return transmission is bidirectional between Ethereum and Litecoin during the pre-COVID-19 period. However, a unidirectional return transmission is observed from Ethereum to Litecoin during the COVID-19 period, implying that Ethereum returns are useful in forecasting Litecoin returns during the COVID-19 period. Overall, the return spillovers vary across the two periods for the pairs of Bitcoin–Ethereum, Bitcoin–Litecoin, and Ethereum–Litecoin.

Regarding volatility spillover, the findings reveal bidirectional and negative volatility transmission between Bitcoin and Ethereum during the pre-COVID-19 period. Moreover, the volatility transmission is positive from Bitcoin to Ethereum, whereas it is negative from Ethereum to Bitcoin during the COVID-19 period. The volatility transmission is unidirectional and negative from Bitcoin to Litecoin during the pre-COVID-19 period, and it is bidirectional during the COVID-19 period. Last, a bidirectional volatility spillover is observed for the pairs of Ethereum–Litecoin during both sample periods. The above-mentioned unidirectional/bidirectional volatility linkages suggest that crypto-investors cannot get the maximum benefit of diversification by making portfolios of these three pairs (i.e., Bitcoin–Ethereum, Bitcoin–Litecoin, and Litecoin–Ethereum). The constant conditional correlations between all pairs of cryptocurrencies are observed to be higher during the COVID-19 period compared to the pre-COVID-19 period, suggesting there are lesser diversification benefits to making portfolios of all pairs of cryptocurrencies during COVID-19.

Based on optimal weights, investors should decrease their investments (a) in Bitcoin for portfolios of BTC/ETH and BTC/LTC, (b) in Ethereum for the portfolio of ETH/LTC during the COVID-19 period. Based on hedge ratios, the optimal hedge ratios are found to be higher for the BTC/ETH, BTC/LTC, and ETH/LTC pairs during the COVID-19 period, which implies that hedging is expensive during the COVID-19 period compared to the pre-COVID-19 period. Finally, a higher hedging effectiveness score shows a higher risk reduction, and our results reveal that the hedging effectiveness is higher during the COVID-19 period compared to the pre-COVID-19 period.

Overall, our findings are not only valuable for understanding of the interrelationships between the major cryptocurrencies, but they are also of great interest to portfolio managers, investors, and investment funds that are actively dealing in Bitcoin, Ethereum, and Litecoin. Indeed, optimal portfolios and hedge ratios are useful for investors in making a portfolio that can reduce risk exposure during both crisis and non-crisis periods. For policymakers, a change in the level of volatility transmission between the major cryptocurrencies implies that the instability of one cryptocurrency can deeply affect the other cryptocurrencies. For instance, any change in Bitcoin would require close monitoring and careful follow-up from policymakers with regard to other cryptocurrencies to avoid adverse consequences from contagious shocks. Overall, these findings provide useful information for portfolio managers and policymakers regarding optimal asset allocation, diversification, hedging, forecasting, and risk management.

## Data Availability

The datasets will be provided on request.

## Appendix

See Tables 6 and 7.

**Table 6:** Break point unit root test

	Test statistic	Break dates
BTC	− 19.981^a^	12/03/2020 23:00
ETH	− 23.479^a^	12/03/2020 10:00
LTC	− 19.850^a^	13/03/2020 01:00

This test is applied on full sample (03/10/2018 to 01/04/2020). Break Selection: Minimize Dickey–Fuller t-statistic

^a^Indicate the statistical significance at 1%, 5% and 10% respectively

**Table 7:** Estimates of tri-variate VAR-AGARCH for the Bitcoin, Ethereum, and Litecoin (Robustness check)

	Pre COVID-19 (01 December 2018 to 31 December 2020)		COVID-19 (13 January 2020 to 01 April 2020)	
	Coefficient	*P* value	Coefficient	*P* value
*Panel A. Mean equation*				
$\mu_{1}$	− 0.000	0.406	0.000	0.142
$\emptyset_{11}$	− 0.134^a^	0.000	− 0.115^b^	0.028
$\emptyset_{12}$	− 0.001	0.908	0.074^c^	0.097
$\emptyset_{13}$	0.113^c^	0.073	0.037	0.569
$\mu_{2}$	− 0.001	0.170	0.000^b^	0.012
$\emptyset_{21}$	0.043^b^	0.016	0.024	0.505
$\emptyset_{22}$	− 0.089^a^	0.001	− 0.182^a^	0.001
$\emptyset_{23}$	0.148^a^	0.000	0.112^b^	0.048
$\mu_{3}$	− 0.000	0.305	0.000	0.317
$\emptyset_{31}$	0.019	0.103	0.026	0.236
$\emptyset_{32}$	0.032^c^	0.060	0.011	0.745
$\emptyset_{33}$	− 0.196^a^	0.000	− 0.258^a^	0.000
*Panel B. Variance equation*				
$c_{1}$	0.001^a^	0.000	0.000^a^	0.000
$c_{2}$	0.000^b^	0.016	0.000^a^	0.000
$c_{3}$	0.000^b^	0.032	0.001^a^	0.001
$a_{11}$	0.124^a^	0.001	− 0.022^c^	0.094
$a_{12}$	0.011^b^	0.032	0.079^a^	0.000
$a_{13}$	0.019^b^	0.013	− 0.003^c^	0.086
$a_{21}$	0.019	0.115	− 0.072^a^	0.000
$a_{22}$	0.031^c^	0.051	0.186^a^	0.000
$a_{23}$	0.014^b^	0.033	0.012^c^	0.053
$a_{31}$	0.018	0.548	− 0.108^a^	0.002
$a_{32}$	0.001	0.736	0.172^a^	0.003
$a_{33}$	0.155^a^	0.001	0.059^b^	0.050
$b_{11}$	0.864^a^	0.000	0.986^a^	0.000
$b_{12}$	− 0.036^b^	0.027	0.016^b^	0.042
$b_{13}$	− 0.022	0.290	0.075^a^	0.000
$b_{21}$	− 0.024^c^	0.075	0.028^b^	0.033
$b_{22}$	0.953^a^	0.000	0.885^a^	0.000
$b_{23}$	− 0.010	0.135	− 0.060^a^	0.000
$b_{31}$	− 0.035	0.443	0.028	0.169
$b_{32}$	0.277^c^	0.099	0.115^c^	0.084
$b_{33}$	0.636^a^	0.000	0.522^a^	0.000
$d_{1}$	− 0.052^b^	0.050	0.041	0.070
$d_{2}$	− 0.018^b^	0.025	0.030^c^	0.093
$d_{3}$	− 0.103^a^	0.000	0.064^b^	0.032
*Panel C: Constant correlations*				
$p_{21}$	0.783^a^	0.000	0.869^a^	0.000
$p_{31}$	0.658^a^	0.000	0.803^a^	0.000
$p_{32}$	0.710^a^	0.000	0.831^a^	0.000
*Panel D: Robustness tests*				
Log L	10,711.7		18,493.2	
AIC	− 21.416		− 20.046	
SIC	− 21.268		− 19.467	
$Q_{1}$(20)	31.675^b^	0.046	33.334^b^	0.030
$Q_{2}$(20)	39.436^a^	0.005	33.455^b^	0.030
$Q_{3}$(20)	25.676	0.176	22.340	0.322
$Q_{1}^{2}$(20)	2.916	0.997	14.670	0.672
$Q_{2}^{2}$(20)	4.055	0.989	11.778	0.740
$Q_{3}^{2}$(20)	6.238	0.988	16.621	0.802

# of lags for VAR is decided using SIC and AIC criteria. JB, Q(20), and Q^2^(20) indicate the empirical statistics of Jarque–Bera test for normality, Ljung–Box Q statistics of order 20 for autocorrelation applied to the standardized residuals and squared standardized residuals, respectively. BTC, Bitcoin; ETH, Ethereum; LTC, Litecoin. Variable order is the Bitcoin (1), Ethereum (2), and Litecoin (3). In the mean equations, $\mu$ denotes the constant terms, whereas $\emptyset_{12}$ denotes the return spillover from Bitcoin to Ethereum. In the variance equation, 'c' denotes the constant terms, 'a' denotes the ARCH terms, and 'b' denotes the GARCH terms. In the variance equation, $a_{12}$ indicates the shock spillover from Bitcoin to Ethereum, whereas ${\text{b}}_{12}$ denotes the long-term volatility spillover from Bitcoin to Ethereum. $d_{1}$ is the asymmetric effect of the Bitocoin

^a,b,c^Indicate the statistical significance at 1%, 5% and 10% respectively

## Footnotes

1 https://www.businessinsider.com/top-cryptocurrencies.

2 https://www.forbes.com/.

3 https://news.un.org/.

4 It is suggested by reviewer to calculate the time varying correlations and hedge ratios for all pairs of cryptocurrencies.

5 To interpret the asymmetric coefficient, we follow the paper of Sadorsky (2014).

6 It is suggested by a reviewer that we check the robustness of the results after changing the start of pre-COVID-19 and COVID-19 periods.
